# A clinical pilot study on the effect of the probiotic *Lacticaseibacillus rhamnosus* TOM 22.8 strain in women with vaginal dysbiosis

**DOI:** 10.1038/s41598-021-81931-z

**Published:** 2021-01-28

**Authors:** Alessandra Pino, Agnese Maria Chiara Rapisarda, Salvatore Giovanni Vitale, Stefano Cianci, Cinzia Caggia, Cinzia Lucia Randazzo, Antonio Cianci

**Affiliations:** 1 Department of Agricultural, Food and Environment, University of Catania, Santa Sofia Street 100, 95123 Catania, Italy; 2 Department of General Surgery and Medical Surgical Specialties, University of Catania, 95123 Catania, Italy; 3 Department of Obstetrics and Gynecology, University of Messina, Messina, Italy

**Keywords:** Microbiology, Signs and symptoms

## Abstract

Lactobacilli with probiotic features play an essential role in maintaining a balanced vaginal microbiota and their administration has been suggested for the treatment and prevention of vaginal dysbiosis. The present study was aimed to in vitro and in vivo investigate the probiotic potential of the *Lacticaseibacillus rhamnosus* TOM 22.8 strain, isolated from the vaginal ecosystem of a healthy woman. For this purpose, safety and functional properties were in depth evaluated. The strain exhibited a broad spectrum of antagonistic activity against vaginal pathogens; adhesion capacity to both the vaginal VK2/E6E7 and the intestinal Caco-2 cells; anti-inflammatory and antioxidant activities, suggesting its promising probiotic features. In addition, an in vivo pilot-study was planned. Based on both clinical and microbiological parameters, the oral or vaginal strain administration, determined a significant pathogens reduction after 10 days of administration and a maintenance of eubiosis up to 30 days after the end of the treatment. Therefore, the *L. rhamnosus* TOM 22.8 strain can be proposed as valuable oral and/or vaginal treatment for vaginal dysbiosis.

## Introduction

The vaginal microbiota of reproductive age healthy women is dominated by lactobacilli, which play an essential protecting role against genitourinary pathogens^1,2^. It is noteworthy that specific lactobacilli are interesting for use as probiotics, which are defined, by the Food and Agriculture Organization of the United Nations and World Health Organization, as “live microorganisms which when administered in adequate amounts, confers health benefits to the host”^3^. Lactobacilli, with probiotic features, are able to antagonize pathogenic microorganisms through different mechanisms of action such as the production of antimicrobial compounds^4^; the ability to adhere to the vaginal epithelial cells surface, inhibiting the adhesion of pathogens by competition for nutrients, steric exclusion/displacement, biofilm production, and co-aggregation^5^; or stimulation of the immune system via interactions with immune cells^6^. For these reasons, there is a growing interest in the in vitro selection of promising probiotic strains as well as in the in vivo evaluation of their ability to treat and/or prevent vaginal imbalance. Although the usefulness of probiotics administration has long been considered, recently their efficacy has been demonstrated, and, unlike antibiotic treatment, no adverse effects have been reported^7–9^.

To date, several clinical trials have shown that both oral and vaginal probiotic administration hold promise to cure and prevent bacterial vaginosis (BV) and vulvovaginal candidiasis (VVC)^10,11^. Indeed, being the vaginal dysbiosis is caused by several bacterial species, both aerobic and anaerobic, and different *Candida* species, antibiotic treatment is often ineffective. The unsuccessfulness of antibiotics is also related to the widespread antibiotic resistance among pathogens responsible for vaginal imbalance. For these reasons, the validation of alternative or complementary approaches represents a medical priority^8^. In this context, probiotic lactobacilli have been used as adjuvant or maintenance therapy preventing incidence or recurrence of BV and VVC or as a stand-alone dysbiosis treatment. As recently reviewed by Jeng et al.^9^ the administration of lactobacilli, such as *L. rhamnosus BMX54*, *L. fermentum*, *L. plantarum*, *L. gasseri*, *L. plantarum*, *L. acidophilus*, *L. brevis CD2*, *L. salivarius subsp. salicinius*, *L. delbrueckii subsp. lactis*, *L. reuteri*, *L. casei*, *L. reuteri*, *L. crispatus*, and *L. rhamnosus GG*, either alone or in various combinations, had a significant effect in the treatment of common vaginal infections in non-pregnant females^9^. However, the majority of the studies evaluated the vaginally applied probiotics and, given the current evidence, a strain-dependent effect was demonstrated^7,12^. Indeed, the mode, dosing, frequency, and duration of the probiotic administration as well as the target population and recruitment strategy should be defined a priori, for the success of the probiotic treatment in restoring a balanced vaginal microbiota^13,14^.

The aim of the present study was to investigate the in vitro and in vivo probiotic properties of the *L. rhamnosus* TOM 22.8 strain (DSM 33500), isolated from the vaginal ecosystem of a healthy woman. Safety properties, antagonistic activity against pathogens, tolerance to simulate gastrointestinal conditions, adhesion ability, antioxidant and anti-inflammatory properties were in depth evaluated. In addition, a pilot study was conducted in order to in vivo validate the suitability of the *L. rhamnosus* TOM 22.8 strain as a promising stand-alone treatment for both BV and VVC. To define the appropriate probiotic delivery mode, both oral and vaginal administration were evaluated.

## Results

### Safety properties

The *L. rhamnosus* TOM 22.8 strain fulfilled all the tested safety requirements since did not show DNAse, gelatinase, mucin degradation ability, haemolytic and bile salt hydrolase activities (data not shown). As reported in Supplementary Table S1 online, the *L. rhamnosus* TOM 22.8 strain was susceptible to all the antibiotics suggested by the EFSA guidelines whereas resistance to Metronidazole (> 600 µg/ml) and Clotrimazole (> 256 µg/ml) were detected. Regarding to boric acid the *L. rhamnosus* TOM 22.8 strain showed a MIC value > 10,000 µg/ml (Supplementary Table S1 online).

### Tolerance to lysozyme, gastric acidic, bile salts and gastrointestinal transit

Results of the tolerance to lysozyme, acidic conditions, and bile salts as well as survivability during simulated gastrointestinal transit are reported in Table 1. Overall, the *L. rhamnosus* TOM 22.8 strain showed the ability to survive under the stressful conditions occurring in the mouth and during the GI digestion. Regarding the tolerance to lysozyme, the *L. rhamnosus* TOM 22.8 strain showed survival rate (SR%) of 94% and 91% after 30 and 120 min of incubation, respectively. Showing SR% higher than 90%, the tested strain was categorized as lysozyme-resistant based on the classification proposed by Solieri et al.^15^. Tolerance to both pH 2.0 and 3.0 was shown by the tested strain after 2 and 4 h of incubation (Table 1). In fact, starting from an initial number of viable cells of 9.11 log cfu/ml, the *L. rhamnosus* TOM 22.8 strain exhibited SR% higher than 90% for all the tested conditions. The *L. rhamnosus* TOM 22.8 strain revealed the ability to tolerate both 0.5% and 1% of bovine bile salts showing SR% higher than 80% after both 2 h and 4 h of incubation. During simulated GI digestion, survivability higher than 90% was observed after treatment with simulated gastric juice (SGJ) and simulated intestinal fluid (SIF) (Table 1).

**Table 1:** Tolerance to lysozyme, acidic conditions, bile salts and survivability during simulated gastrointestinal transit exhibited by the tested *L. rhamnosus* TOM 22.8 strain.

Tested activity	Condition	Time	Survival rate (SR%)
Lysozyme tolerance		30 min	94
		120 min	91
Tolerance to low pH	3.0	2 h	97
		4 h	93
	2.0	2 h	96
		4 h	92
Tolerance to bile salts	0.5%	2 h	89
		4 h	88
	1%	2 h	88
		4 h	82
Simulated GI digestion	SGJ^a^		99
	SIF^b^		97

Survival rate (SR%) was calculated based on the initial and the final number of viable cells enumerated on MRS agar after 48 h.

^a^Simulated gastric juice.

^b^Simulated intestinal fluid.

### Antimicrobial activity against pathogens

Results of antimicrobial activity against pathogens are reported in Table 2. Overall, the *L. rhamnosus* TOM 22.8 strain showed a broad spectrum of antagonistic activity against all the tested pathogens with the exception of *C. parapsilosis* ATCC 90018. The highest antimicrobial activity was displayed against *E. coli* ATCC 25922, *E. coli* ATCC 700414, and *G. vaginalis* ATCC 14018 stains (inhibition zone larger than 20 mm). Inhibition zones with a diameter between 11 and 20 mm were observed against *G. vaginalis* ATCC 14019, *C. albicans* ATCC10231, *C. krusei* ATCC 14243, *C. glabrata* ATCC 90030, *C. tropicalis* ATCC 13803, and *S. aureus* ATCC 6538 pathogen strains (Table 2).

**Table 2:** Antimicrobial activity against pathogens, surface properties, hydrogen peroxidase (H_2_O_2_) and exopolysaccharide (EPS) production exhibited by the *L. rhamnosus* TOM 22.8 strain.

Antimicrobial activity	
Tested pathogens	TOM 22.8
*E. coli* ATCC 25922	+++
*E. coli* ATCC 700414	+++
*G. vaginalis* ATCC 14018	+++
*G. vaginalis* ATCC 14019	++
*C. albicans* ATCC10231	++
*C. krusei* ATCC 14243	++
*C. glabrata* ATCC 90030	++
*C. tropicalis* ATCC 13803	++
*S. aureus* ATCC 6538	++
*C. parapsilosis* ATCC 90018	−
**Surface properties**	
H%^a^	82.12 ± 0.13
Auto-A%^a^	66.54 ± 0.04
**CoA%**^a^	
*E. coli 555*	61.28 ± 0.07
*G. vaginalis*	67.35 ± 0.15
*C. albicans*	61.24 ± 0.15
*C. glabrata*	65.33 ± 0.09
H_2_O_2_^b^	3
EPS (mg/l)	273

(−) no inhibition zone, (+) inhibition zone < 10 mm; (++) inhibition zone 11–20 mm; (+++) inhibition zone > 20 mm.

^a^Results of surface properties (H%: hydrophobicity; auto-A%: auto-aggregation; CoA%: co-aggregation) are expressed as average percentage value and standard deviation of three separate experiments.

^b^Results of H_2_O_2_ production was scored as: 0, (absent, no production of blue coloration); 1 (low production, time > 20 min), 2 (medium production, time 10–20 min), and 3 (high production, time < 10 min).

### Preliminary identification of metabolites responsible for antagonistic activity against pathogens

To determine the nature of the substances (e.g., bacteriocins, organic acids, and/or hydrogen peroxide) responsible for the antimicrobial activity exhibited by the TOM 22.8 strain against pathogens, the cell free supernatant (CFS) was differently treated. In detail, the treatment of CFS with proteases did not result in any changes of antimicrobial activity, indicating that the antagonism was not due to bacteriocins production or protein-based compounds. Differently, after treating the CFS with catalase and after CFS neutralization, no antagonistic activity against pathogens was showed by the tested strain, suggesting that both hydrogen peroxides and organic acids production were probably responsible for the antagonistic activity.

### Hydrophobicity, auto-aggregation and co-aggregation abilities; hydrogen peroxide and exopolysaccharide production

Results of surface properties (hydrophobicity, auto-aggregation and co-aggregation), hydrogen peroxide and exopolysaccharide production abilities exhibited by the *L. rhamnosus* TOM 22.8 strain are reported in Table 2. The tested strain showed cell surface hydrophobicity of 82.12% whereas the auto-aggregation percentage was 66.54%. Overall, the *L. rhamnosus* TOM 22.8 strain showed co-aggregation abilities with all the tested pathogens with values ranging from 61.24 to 67.35% (Table 2). Regarding the hydrogen peroxide production, the tested strain was classified as high producer (score 3) since the time needed to the appearance of blue coloration was less than 10 min. The *L. rhamnosus* TOM 22.8 strain was able to produce EPS with a value of 273 mg/l (Table 2).

### In vitro adhesion assay

The adhesion abilities to Caco-2 and to VK2/E6E7 vaginal epithelial cells of the tested lactobacilli strains, are shown in Table 3. Overall, the adhesion capacity was strain-dependent. The *L. rhamnosus* TOM 22.8 strain exhibited the highest binding ability to both Caco-2 and VK/E6E7 cells.

**Table 3:** Gene levels expression of COX-1, COX-2, IL-8, and IL-10 in differentiated human macrophages and adhesion (%) of lactobacilli to Caco-2 and to VK2/E6E7 vaginal epithelial cells.

	Anti-inflammatory activity				Adhesion %	
	COX-1	COX-2	IL-8	IL-10	Caco-2	VK2/E6E7
LPS	2.11 ± 0.10	15.23 ± 0.76	0.67 ± 0.03	125.50 ± 6.42		
***Lacticaseibacillus rhamnosus***						
TOM 22.8	23.15 ± 1.26*	0.06 ± 0.03*	0.54 ± 0.05	611.83 ± 1.32*	14.21 ± 0.07	12.1 ± 0.11
E21	2.03 ± 0.2	14.91 ± 0. 13	0.78 ± 0.05	123.74 ± 1.38	12.6 ± 0.18	10.87 ± 0.20
L3	2.15 ± 0.18	14.96 ± 0.24	0.73 ± 0.08	124.38 ± 1.24	11.4 ± 0.16	10.2 ± 0.13
***Lactobacillus helveticus***						
P7	2.21 ± 0.11	15.03 ± 0.21	0.65 ± 0.14	123.28 ± 1.42	10.9 ± 0.12	11.5 ± 0.05
P12	2.14 ± 0.33	15.09 ± 0.18	0.75 ± 0.23	125.78 ± 1.11	7.14 ± 0.09	8.41 ± 0.16
S7	2.00 ± 0.51	15.11 ± 0.14	0.61 ± 0.27	124.71 ± 1.04	9.12 ± 0.11	6.38 ± 0.09
U13	2.06 ± 0.28	15.20 ± 0.22	0.67 ± 0.21	124.96 ± 0.93	7.64 ± 0.23	8.9 ± 0.13
***Ligilactobacillus salivarius***						
N30	2.13 ± 0.05	16.01 ± 0.10	0.66 ± 0.08	124.91 ± 0.81	8.13 ± 0.16	9.16 ± 0.18
***Lacticaseibacillus rhamnosus***						
GG	nt	nt	nt	nt	13.5 ± 0.09	12.3 ± 0.16

Adhesion to Caco-2 and to VK2/E6E7 vaginal epithelial cells are expressed as percentage (%). The adhesion abilities of the E21, L3, P7, P12, S7, U13, and N30 strains was already published^23^.

COX-1,COX-2,IL-8, and IL-10 data are expressed as increased fold compared to untreated cells and standard deviation; *nt* not tested.

*P < 0.05 respect to lipopolysaccharide (LPS) treatment.

### In vitro antioxidant and anti-inflammatory activities

**Figure 1:**
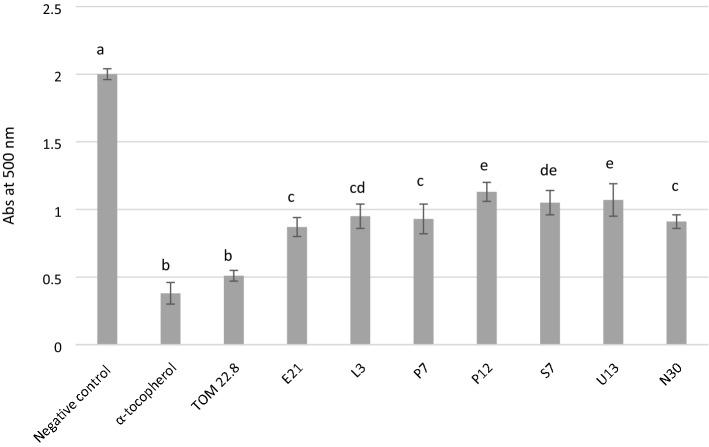
Lipid peroxidation inhibitory activity (Abs at 500 nm) of the tested lactobacilli strains. The activity was measured under a linoleic acid oxidation system for 8 days. α-Tocopherol (1 mg/ml) was used as positive control. Data are the means from three independent experiments. Bars represent standard deviations. Letters a–e indicate P < 0.05.

Antioxidant abilities exhibited by the tested strains are reported in Fig. 1. The *L. rhamnosus* TOM 22.8 strain was able to counteract the peroxidation of linoleic acid showing antioxidant activity similar (P > 0.05) to those detected for the α-tocopherol.

Table 3 shows the anti-inflammatory activity of the tested lactobacilli. The *L. rhamnosus* TOM 22.8 strain, was able to downregulate the gene expression of COX-2 and to determine the upregulation of IL-10. In addition, the reduction of the expression levels of the IL-8 gene was detected (Table 3).

### Capsules stability testing

The capsules stability during the 18-months storage at 25 °C ± 2 °C under 60% ± 5% of humidity revealed that the product was compliant with regulatory specification showing the absence of *E. coli*, *Salmonella* spp., and *Staphylococcus aureus*. In addition, Gram negative bile tolerant, total yeast and mould, and total aerobic microbial counts were lower than 1 log unit. High stability of the *L. rhamnosus* TOM 22.8 encapsulated strain was detected showing cell density of 10 log cfu/g after 18 months of storage (data not shown).

### Demographic and clinical findings of patients recruited in the pilot study

Overall, 30 women, aged between 18 and 45 years (mean age of 36.73 ± 5.96 years), satisfying the inclusion and exclusion criteria, were enrolled in the pilot study. The demographic and clinical characteristics of the enrolled patients are shown in Table 4.

**Table 4:** Demographic and clinical characteristics of the study sample (n = 30).

**Demographic characteristics (n = 30)**	
Age	36.73 ± 5.96
Sexual activity	26 (86.67%)
Smoking	8 (26.67%)
Body mass index (kg/m^2^)	23.02 ± 1.87
< 18.5	1 (1.43%)
18.5–24.9	56 (80%)
25–29.9	8 (11.43%)
≥ 30	5 (7.14%)
Contraceptive use	23 (77%)
Oral	5 (22%)
Barrier	8 (35%)
Others	10 (43%)
**Clinical and microbiological characteristics (n = 30)**	
Vulvovaginal signs and symptoms	
Leucorrhoea	30 (100%)
Burning	15 (50%)
Itching	13 (43.33%)
Subjective vulvar discomfort	30 (100%)
Amsel criteria	
Homogenous vaginal discharge	27 (90%)
Clue cell presence	16 (53.55%)
Positive amine test	30 (100%)
Vaginal pH > 4.5	30 (100%)
Nugent score	
0–3	0
4–6	0
7–10	30 (100%)
Lactobacillary grade	
1	0
2	2 (6.66%)
3	28 (93.33%)

Data are presented as both absolute number and percentages*.* Age and body mass index are expressed as mean and standard deviation.

Participants’ self-reported signs or symptoms during the whole study are shown in Supplementary Fig. S1 online. At the baseline evaluation, all the examined women (100%) reported leucorrhoea and subjective vulvar discomfort, while mild symptoms of burning and itching were reported in 50% and 43% of cases, respectively.

Both A and B groups (vaginal and oral administration of the *L. rhamnosus* TOM 22.8 strain, respectively) showed a statistically significant improvement of self-reported symptoms at both T1 (10 days after the start of the administration) and T2 (30 days after the end of the administration) sampling times. On the contrary, during the first follow-up visit no improvement of clinical conditions was observed in patients subjected to a wait and see approach (group C), while a slight worsening of some self-reported symptoms was observed at the T2 follow up.

For each group, results of clinical criteria evaluation at baseline (T0), 10 days after the start of the treatment (T1), and 30 days after the end of the treatment (T2) are shown in Table 5. At the baseline assessment, all the examined women had at least 3 Amsel criteria, a Nugent score ≥ 7 or a Lactobacillary grade ≥ 2. Both A and B groups showed, at T1 and T2 sampling time, an improvement of all the clinical criteria taken into account. In fact, a statistically significant change of Amsel criteria and a reduction of Nugent score were achieved. In addition, the improvement of the Lactobacillary grade was detected during the whole pilot-study (Table 5). Different trend was observed in group C, which showed no symptoms improvement although no significant worsening was reported.

**Table 5:** Clinical criteria evaluation for each treatment group (A: vaginal administration, B: oral administration; C: wait and see) at baseline (T0), 10 days after the start of the treatment (T1) and 30 days after the end of the treatment (T2).

Diagnostic parameters	Group A (vaginal)				Group B (oral)				Group C (wait and see)			
	T0	T1	T2	P*	T0	T1	T2	P*	T0	T1	T2	P*
**Amsel criteria**												
Homogenous vaginal discharge	10	3	1	< 0.05	9	1	0	< 0.05	8	9	10	0.329
Clue cell presence	3	0	0	0.036	7	0	0	< 0.05	6	7	7	0.861
Positive amine test	10	1	1	< 0.05	10	0	0	< 0.05	10	9	9	0.585
Vaginal pH > 4.5	10	1	1	< 0.05	10	0	3	< 0.05	10	10	10	–
**Nugent score**												
0–3	0	8	8	< 0.05	0	10	10	< 0.05	0	0	0	–
4–6	0	2	2	0.315	0	0	0	–	0	0	1	0.355
7–10	10	0	0	< 0.05	10	0	0	< 0.05	10	10	9	0.355

*Statistical significance P < 0.05; P refers to all pairwise comparisons.

No adverse events were recorded in either study group for all the entire observational period and nobody among participants was excluded from the study due to the onset of adverse events.

### Microbial counts of vaginal swabs collected during the in vivo trial

Microbial counts, expressed as mean and standard deviation of log cfu/ml of the main microbial groups detected during the in vivo trial, are reported in Table 6. At baseline (T0), all participants had an imbalanced microbiota dominated by potentially pathogenic bacteria with reduced cell density of lactobacilli. After 10 days (T1) of vaginal (group A) or oral (group B) administration of the potential probiotic *L. rhamnosus* TOM 22.8 strain, a significant reduction of all the pathogenic bacteria was observed. On the contrary, the level of lactobacilli significantly increased (P < 0.05) of about 3 log units. Thirty days after the end of the treatment (T2) the vaginal microbiota composition was quite stable and similar to those observed at T1 sampling time.

**Table 6:** Microbiological analysis and ANOVA significance of vaginal discharge collected at baseline (T0), after 10 days of the *L. rhamnosus* TOM 22.8 strain administration (T1) and 1 month after the *L. rhamnosus* TOM 22.8 strain administration (T2).

Microbial groups	Oral administration						Vaginal administration						Wait and see					
	T0	T1	T2	*P T0 vs T1	*P T0 vs T2	*P T1 vs T2	T0	T1	T2	*P T0 vs T1	*P T0 vs T2	*P T1 vs T2	T0	T1	T2	*P T0 vs T1	*P T0 vs T2	*P T1 vs T2
*Lactobacilli*	3.85 ± 0.22	6.95 ± 0.19	8.23 ± 0.12	0.0001	1.71 × 10^–5^	0.001178	2.95 ± 0.25	5.80 ± 0.17	5.90 ± 0.11	0.0002	0.0001	0.5305	4.02 ± 0.71	4.18 ± 0.87	3.87 ± 0.89	0.6638	0.7138	0.4666
*Enterococcus* spp.	1.70 ± 0.16	0.52 ± 0.21	0.51 ± 0.03	0.0017	0.000395	0.698066	< 1	< 1	< 1	–	–	–	4.14 ± 0.77	4.38 ± 0.94	4.78 ± 0.94	0.5611	0.1359	0.3856
*Staphylococcus* spp.	< 1	< 1	< 1	–	–	–	< 1	< 1	< 1	–	–	–	2.11 ± 1.69	2.37 ± 1.59	2.42 ± 1.83	0.7372	0.7120	0.95334
*Streptococcus* spp.	< 1	< 1	< 1	–	–	–	< 1	< 1	< 1	–	–	–	4.07 ± 1.32	4.23 ± 0.98	4.61 ± 1.15	0.7719	0.3693	0.4657
*Gardnerella vaginalis*	4.20 ± 0.21	1.06 ± 0.33	1.03 ± 0.09	0.0004	4.11 × 10^–5^	0.970612	< 1	< 1	< 1	–	–	–	4.27 ± 0.36	4.34 ± 0.37	4.40 ± 0.27	0.6582	0.3739	0.69789
*Candida glabrata*	5.60 ± 0.22	1.51 ± 0.24	1.32 ± 0.06	5.60 × 10^–5^	9.98 × 10^–6^	0.386837	4.16 ± 0.21	0.8 ± 0.22	0.12 ± 0.03	0.0001	1.19 × 10^–5^	0.0103	3.61 ± 1.52	3.85 ± 1.74	4.04 ± 1.77	0.7637	0.5883	0.8183
*Candida albicans*	3.40 ± 0.21	0.27 ± 0.31	0.11 ± 0.03	5.53 × 10^–5^	2.47 × 10^–5^	0.174456	4.81 ± 0.20	1.2 ± 0.29	1.28 ± 0.01	0.0001	1.79 × 10^–5^	0.7297	3.65 ± 1.30	3.78 ± 1.37	3.92 ± 1.42	0.8479	0.6826	0.8291
*Candida krusei*	3.74 ± 0.18	1.24 ± 0.10	1.14 ± 0.16	2.93 × 10^–5^	6.28 × 10^–5^	0.399079	4.41 ± 0.15	1.03 ± 0.08	1.06 ± 0.13	9.07 × 10^–6^	1.75 × 10^–5^	0.7966	2.90 ± 1.51	3.62 ± 1.91	3.64 ± 1.91	0.3849	0.3726	0.9834
*Escherichia coli*	3.30 ± 0.28	1.07 ± 0.14	1.05 ± 0.09	0.0005	0.000413	0.872158	< 1	< 1	< 1	–	–	–	1.83 ± 1.26	2.50 ± 1.50	2.68 ± 1.52	0.3178	0.2127	0.8051

Data are shown as mean and standard deviation.

*Statistical significance P < 0.05.

Different trend was achieved for patients allocated in the wait and see group (group C). In fact, high cell density of the pathogenic bacteria and low lactobacilli count was observed at each sampling time (T0, T1, and T2). In addition, no significant changes in the vaginal microbiota composition was detected during the whole study.

## Discussion

Nowadays, the understanding of the vaginal microbiota balance and the concern about the treatment of dysbiosis have highlighted the promising role of probiotics as a new therapeutic or adjuvant treatment for vaginal microbiota homeostasis^10,11^.

Albeit a reasonable number of probiotic strains have been characterized, in recent years, the regulatory *scenario* for human probiotics has changed considerably. Notably, both the scientific community and industry have gained increasing interest in the identification of new strains with specific health-promoting properties. According to that, the present study was aimed to in vitro evaluate the probiotic potential of the *L. rhamnosus* TOM 22.8 strain and to in vivo validate its ability to restore a balanced vaginal microbiota. The aforementioned strain derived from a total of 400 vaginal isolates; 261 of which were previously screened^16^; among the remaining 139, the *L. rhamnosus* TOM 22.8 strain was in depth investigated in clinical evidence-based product development.

As established by the FAO/WHO guidelines^3^, the fulfilment of safety properties is mandatary for probiotic strains. In this context, one of the main concerns is related to the spread of antibiotic resistance by horizontal transfer of genes among bacteria^17,18^. Therefore, promising probiotic strains should not carry transferable antibiotic resistance. In this study, the antibiotics susceptibility of the *L. rhamnosus* TOM 22.8 strain was evaluated according to the European Food Safety Authority (EFSA)^19^ guidelines and as a result, no antibiotic resistance was detected. In addition, the antibiotics susceptibility of the strain was also tested *versus* the most routinely used antimicrobials for the treatment of BV and VVC. It is interesting to highlight the high resistance of the strain to metronidazole, clotrimazole and boric acid, suggesting its possible use as adjuvant to the antibiotic treatment, in reducing BV and VVC recurrence. In order to orally administrate the *L. rhamnosus* TOM 22.8 strain, resistance to salivary enzymes, stomach acidity and to the antimicrobial action of pepsin, which acts as a defence barrier^20^, were addressed. In addition, promising probiotic strains should be able to colonize the digestive tract epithelium cells, preventing the exclusion by peristalses as well as inhibiting the adhesion of pathogens to intestinal and/or vaginal epithelium^16,21–23^. According to that, our data revealed that the *L. rhamnosus* TOM 22.8 strain was able to survive throughout the GI tract passage during the simulated GI digestion, to adhere to the epithelium surface and through the co-aggregation ability to create a microenvironment hostile to the adhesion of pathogens. In this regard, the aforementioned strain showed a broad range of antagonistic activity against the pathogens responsible for vaginal dysbiosis, including those dominated by *Candida* spp., in accordance to previously reported data^24–26^.

It is important to underline that in clinical practice, *Candida* infection is often associated to other microorganisms, even in presence of increased vaginal pH, indicating that mixed infections of *Candida* with bacterial vaginosis, aerobic vaginitis, or both are not infrequent^7^. These clinical pictures are frequently paucisymptomatic and should be differentiated by acute forms of candidiasis, which are usually accompanied by the occurrence of relevant symptoms^27^. Moreover, the interaction of *Candida* with vaginal bacteria can influence the development and the severity of the symptoms. According to that, in the present study, all the enrolled patients showed a mixed vaginal microbiota characterized by the presence of both aerobic and anaerobic bacteria as well as by the coexistence of different *Candida* species.

Although the cause of BV has not been fully elucidated, most studies highlighted that BV is not caused by exogenous infection but by an endogenous imbalance. Moreover, this proposed mechanism supports the assumption that abnormalities of the vaginal flora often persist, even in the absence of significant bothers, and more often appearing a paucisymptomatic form, leading the woman to underestimate and to considered normal these symptoms^28–30^. On the other hand, it is recognised that in circumstances of imbalance of the vaginal ecosystem there is a higher risk for the development of sexually transmitted infections and for adverse reproductive outcomes^31,32^.

It is well established that among probiotic bacteria, lactobacilli exhibit antagonistic knacks against BV and VVC; however, not all lactobacilli strains have demonstrated to exert the desired effects in restoring physiological conditions. Poor colonization of some strains in the vagina^33–35^, inadequate dose and treatment duration could be the reasons. Up to now, no definitive conclusion has been reached on what the best use of probiotics should be: as the main treatment in the absence of antibiotic treatment, as an adjuvant treatment after conventional antibiotics, as a maintenance therapy to prevent recurrences, or as a combination of the aforementioned^11^. Another weakness is related to the choice of the route of administration. Although vaginal administration of lactobacilli is still considered the elective way to treat vaginal disbyiosis, a recent systematic review^11^ has pointed out that none of the 22 vaginal probiotic lactobacilli evaluated was able to colonise the vagina, suggesting that more rigorously future trials are needed to draw firm conclusions.

For this reason, the main goal of this study was to compare the vaginal administration of *L. rhamnosus* TOM 22.8 strain to oral one. It is noteworthy that probiotics orally administrated can ascend from the rectum to the vagina and, even if the treatment time needed to balance the vaginal microbiota homeostasis seems longer than vaginal administration, the oral approach presents some advantages. In fact, the oral lactobacilli administration could reduce the transfer of yeast and pathogenic bacteria from the rectum to the vagina, lowering the risk of BV or/and VVC recurrences^36^.

In the present study, based on symptoms and microbiological counts, both administration routes had a significant impact on the restoration of the vaginal eubiosis condition. Indeed, oral and vaginal treatments were effective in reducing discharge, lowering vaginal pH, and reducing the presence of clue cells, resulting in an effective restoration of the physiological condition, accompanied by the remission or attenuation of clinical signs and symptoms. Notably, the vaginal eubiosis condition was maintained till the second follow-up visit (T2) indicating a significant control of recurrences. Moreover, microbiological data demonstrated that the *L. rhamnosus* TOM 22.8 strain administration significantly reduced the viable cells of all the pathogens investigated, including *Candida* spp. and anaerobic bacteria. On the contrary, the wait and see group showed a substantial maintenance of clinical and microbiological conditions at the first follow-up visit (T1) and a mild clinical worsening at T2 follow-up. Indeed, transient fluctuations over a woman's lifetime usually happen and they are more likely to be the results of daily life activities and behaviours as well as the effect of hormonal fluctuation during the menstrual cycle, rather than the expression of a progressive worsening of the underlying conditions. Although with certain shortcomings in terms of population size and absence of a control group subjected to conventional antibiotic treatment, the present pilot-study demonstrated that the *L. rhamnosus* TOM 22.8 strain administration could be considered as a side effect-free treatment useful to restore a normal vaginal microbiota. Based on our knowledge, no previous studies have compared the effect of both oral and vaginal probiotic in the treatment on vaginal dysbiosis taking into account both clinical (Amsel) and microbiological (Nugent score and pathogen count vs. lactobacilli count) criteria. Based on the aforementioned evidences, the present study suggests the suitability of both vaginal and oral administration of the *L. rhamnosus* TOM 22.8 strain, confirming the evidence that oral intake of probiotics can support the colonization of the vaginal mucosa^33^, restoring the healthy microbiota ecosystem.

Further studies will be done in order to characterize the organic acids (such as lactic, butyric, acetic, citric, succinic, glutamic acids) responsible for the antagonistic activity against pathogens. In addition, clinical studies on a larger number of subjects and with an extension of the follow-up period are needed in order to validate the aforementioned clinical and microbiological findings. Given the promising results that have emerged in the present study, the authors aim to address all these aspects through future investigations.

## Methods

### Isolation of *Lacticaseibacillus rhamnosus* TOM 22.8

The *L. rhamnosus* TOM 22.8 strain belonged to the collection of the Department of Agricultural, Food and Environment, University of Catania, Italy. The strain was previously isolated from the vaginal ecosystem of asymptomatic pre-menopausal Italian women, during a routine gynecological consultation at the Obstetrics and Gynecology Department, General Hospital G. Rodolico, University of Catania (Italy)^16^. The *L. rhamnosus* TOM 22.8 strain derived from a set of 400 isolates, 261 of which were previously screened for safety and functional features^16^. The *L. rhamnosus* TOM 22.8 strain was selected for its interesting probiotic properties among the remaining 139 isolates (unpublished data). The strain was purified three times, microscopically examined, and tested for catalase activity and Gram reaction before storing at − 80 °C in liquid culture, using 20% of glycerol.

### Taxonomic identification at species and strain level

The *L. rhamnosus* TOM 22.8 strain was genetically characterised through whole-genome sequencing performed with an Illumina MiSeq Sequencing System. Genome assemblies of *L. rhamnosus* TOM 22.8 genomes was performed with SPAdes v3.14.0 by means of MEGAnnotator pipeline.

Open reading frames prediction was performed with Prodigal v2.6. Automatic annotation of the ORFs was performed with RAPSearch2 against NCBI RefSeq database and HMMER against PFAM database. Ribosomal RNA genes prediction was performed with RNAmmer v1.2. Transfer RNA genes prediction was performed with tRNAscan-SE v1.21.

In addition, the strain typing was performed through Pulsed Field Gel Electrophoresis (PFGE). In detail, agarose-embedded cells plugs preparation, DNA extraction and enzymatic restriction digestion were carried out using the CHEF Bacterial Genomic DNA Plug Kits (Bio-Rad, UK) according to the protocol indicated by the manufacturer for Gram-positive bacteria. Restriction enzyme digestion was performed with *Asc*I at the 37 °C for 18 h. Electrophoresis was performed using the CHEF DR II system (Bio-Rad, UK) in 1% Megabase agarose (Bio-Rad, UK) in 0.5X TBE buffer. The pulse time was 1.0–20.0 s, the applied current was 6 V/cm, the temperature was 14 °C, and the running time was 17.5 h. The gels were then stained with 1X Atlas ClearSight DNA Stain (BIOATLAS OÜ, EW) solution for one hour and visualised under UV light.

The *L. rhamnosus* TOM 22.8 strain was deposited in the Deutsche Sammlung von Mikroorganismen und Zellkulturen (DSMZ) collection (deposit number DSM 33500).

### Reference strains and culture conditions

The *Streptococcus pyogenes* ATCC 19615 and *Streptococcus pneumoniae* ATCC 6303 strains were cultured on Brain–Heart Infusion (BHI, Becton Dickinson GmbH, Germany) at 37 °C under 5% CO_2_ and used as β-hemolysis and α-hemolysis positive controls, respectively. The *Lactobacillus acidophilus* DRU, used as a positive control for the bile salt hydrolase assay, the hydrogen peroxide (H_2_O_2_) producer *L. acidophilus* ATCC 4356, were routinely cultured in de Man Rogosa and Sharpe (MRS, Biolife, Italy) medium added with 100 mg/l of cycloheximide (Merck, Germany) and incubated at 37 °C under anaerobic conditions, using Anaerocult C (Merck, Milan, Italy). The *L. rhamnosus* GG strain, along with the promising probiotic *L*. *rhamnosus* E21 and L3 strains, *Lactobacillus helveticus* P7, P12, S7, and U13 strains, and *Ligilactobacillus salivarius* N30 strain, previously selected based on both safety and functional features^16^, were used as reference strains for the in vitro adhesion assay. The aforementioned lactobacilli were cultured as reported above.

*Escherichia coli* ATCC 25922 and *Escherichia coli* ATCC 700414 were routinely cultured on Trypticase Soy Broth medium (Oxoid, Milan) at 37 °C under aerobic conditions; *Gardnerella vaginalis* ATCC 14018 and *Gardnerella vaginalis* ATCC 14019 were cultured on Casman’s medium base added of 5% of rabbit blood (VWR, Milan, Italy) at 37 °C; *Candida albicans* ATCC 10231, *Candida krusei* ATCC 14243, *Candida glabrata* ATCC 90030, *Candida parapsilosis* ATCC 90018, and *Candida tropicalis* ATCC 13803 were cultured on Yeast Mold Broth (Conda, Madrid, Spain) at 28 °C under aerobic conditions. All the aforementioned strains were used as reference pathogens for the antagonistic activity assay.

### Safety properties evaluation

The *L. rhamnosus* TOM 22.8 strain was evaluated for DNAse, gelatinase, mucin degradation ability, haemolytic and bile salt hydrolase activities, and antibiotic susceptibility according to Pino et al.^16^. *S. pyogenes* ATCC 19615 and *S. pneumoniae* ATCC 6303 were used as β-hemolysis and α-hemolysis positive controls, respectively. The strain *L*. *acidophilus* DRU was used as bile salt hydrolase positive control.

Antibiotics susceptibility was investigated determining the minimum inhibitory concentration (MIC) by micro-dilution method, following the protocol described by Russo et al.^37^. Resistance or sensitivity to each tested antibiotic was recorded based on the breakpoints proposed by the European Food Safety Authority (EFSA)^19^. In addition, the resistance or sensitivity to boric acid, metronidazole, and clotrimazole was investigated. All the assays were performed in triplicate.

### In vitro assessment of tolerance to lysozyme, acidic conditions and bile salts

Lysozyme tolerance was tested after 0, 30, and 120 min by viable cell counts in MRS agar following the method previously described^38^. Sterile electrolyte solution without lysozyme was used as control.

Tolerance to acidic conditions was evaluated in MRS broth adjusted to pH 2.0 and 3.0 by adding 1 M HCl. MRS broth at pH 6.2 was used as control. Aliquots were collected after 0, 2, and 4 h of incubation at 37 °C and viable cells count was performed on MRS agar plates after incubation at 37 °C for 48 h.

The bile salt tolerance assay was carried out in MRS broth containing 0.5% and 1.0% of bovine bile salts (Oxgall; St. Louis, MO, USA, Sigma-Aldrich). MRS both without bovine bile salts was used as control. After incubation at 37 °C up to 4 h, tenfold serial dilutions were made and plated on MRS agar then incubated at 37 °C for 24 h.

Each assay was performed in triplicate using 9 log cfu/mL bacterial suspension of the *L. rhamnosus* TOM 22.8 strain previously cultured twice in MRS broth. Results were expressed as survival rate percentage (SR %) based on the initial and the final number of viable cells.

### Survivability during simulated gastrointestinal transit

The ability of the *L. rhamnosus* TOM 22.8 strain to survive during the in vitro gastrointestinal transit was evaluated on simulated gastric juice (SGJ) and simulated intestinal fluid (SIF) as reported by Pino et al.^16^.

The assay was performed in triplicate using 9 log cfu/mL bacterial suspension of the *L. rhamnosus* TOM 22.8 strain previously cultured twice in MRS broth. Results were expressed as survival rate percentage (SR %), based on the initial and the final number of viable cells.

### Antimicrobial activity against pathogens

The antagonistic activity of the *L. rhamnosus* TOM 22.8 strain against *E. coli* ATCC 700414, *E. coli* ATCC 25922, *G. vaginalis* ATCC 14018, *G. vaginalis* ATCC 14019, *C. albicans* ATCC 10231, *C. krusei* ATCC 14243, *C. glabrata* ATCC 90030, *C. tropicalis* ATCC 13803, *C. parapsilosis* ATCC 90018, and *S. aureus* ATCC 6538 was tested by the agar spot test following the method described by Pino et al.^16^. Based on the diameter size of the inhibition zones, the antagonistic activity was scored as: (−) no inhibition zone; (+) inhibition zone < 10 mm; (++) inhibition zone between 11 and 20 mm; (+++) inhibition zone > 20 mm.

### Preliminary identification of metabolites responsible for antagonistic activity against pathogens

To elucidate the nature of substances responsible for the antagonistic activity against pathogens, cell-free supernatant (CFS) of the TOM 22.8 strain was obtained by centrifugation of overnight cultures (7000 × *g*, 15 min, 5 °C) and further sterilization by filtration through a 0.22-µm pore filter (Millipore, Italy). CFSs were differently treated according to Pino et al.^39^. The antagonistic activity was evaluated by measuring the diameter of inhibition zones and was determined in triplicate.

### Hydrophobicity, auto-aggregation and co-aggregation abilities

Cell surface hydrophobicity, auto-aggregation and co-aggregation abilities were tested following the protocol described by Pino et al.^16^. In co-aggregation assay *E*. *coli* 555, *G*. *vaginalis* ATCC 14018, *C*. *albicans* ATCC 10231, and *C*. *glabrata* ATCC 90030 were used as pathogenic strains. Results were expressed as percentage (%) for all the assays.

### Hydrogen peroxide and exopolysaccharide production

The ability to produce hydrogen peroxide (H_2_O_2_) was evaluated as reported by Pino et al.^16^ and scored, based on the time needed to the appearance of blue coloration, as: absent (score 0, no production of blue coloration); low (score 1, time > 20 min), medium (score 2, time 10–20 min) and high (score 3, time < 10 min) H_2_O_2_ producer. The *Lactobacillus acidophilus* ATCC 4356 strain was used as positive control.

The production of exopolysaccharide was evaluated following the phenol/sulphuric acid method^40^ and the produced amount, expressed as mg/l, was estimated using glucose (50–500 mg/l) as standard^41^.

### In vitro adhesion assay

Adhesion capacity to both the vaginal VK2/E6E7 (ATCC-CRL-2616) and the intestinal Caco-2 (ATCC HTB-37) epithelial cells was tested according to Pino et al.^16^. The adhesion ability was expressed as percentage and was calculated comparing the number of adherent cells to the initial viable count of the added bacterial suspension (7 log cfu/ml).

### In vitro antioxidant and anti-inflammatory activities

The antioxidant activity of the *L. rhamnosus* TOM 22.8 strain, along with the promising probiotic strains *L. rhamnosus* E21 and L3, *L. helveticus* P7, P12, S7, U13, and *L. salivarius* N30^16^, were tested thought the 2,2-azino di-(3-ethylbenzthiazoline sulfonate) ABTS assay according to Pino et al.^39^.

The anti-inflammatory activity explicated by the tested strain was evaluated using the human pro-monocytic U937 cell line pre-treated with lipopolysaccharide (LPS) in order to induce the inflammation and following the protocol reported by Pino et al.^39^. Quantitative real-time PCR (qRT-PCR) was performed in order to evaluate the gene expression of COX-1, COX-2, IL-8, and IL-10 using the primer pairs and conditions previously described^39^. Untreated cells were used as control. The analyses were performed in triplicate and results are reported as mean and standard deviation.

### Capsules preparation and stability testing

The *L. rhamnosus* TOM 22.8 strain was freeze-dried and subjected to encapsulation using the MG2 Supreme Model Capsule Machine (MG2, Bologna, Italy). Each capsule contained 10 log cfu/g of the *L. rhamnosus* TOM 22.8 strain, dried starch (118.9 mg), magnesium stearate (4.05 mg), and silicon dioxide (4.05 mg). The capsules were produced under carefully controlled conditions. Controls were performed continuously throughout the process to guarantee the compliance to the highest quality standards as defined in the current edition of the Capsugel "Technical Reference File" for Vcaps Plus capsules. The product was conformed to the established A.Q.L.'s for Physical Attributes and was free of preservatives, ethylene oxide treatment, and irradiation treatment. Stability testing was quarterly performed for 18 months to provide evidence of how the quality of active ingredients varies over time under the influence of both temperature and humidity. For this purpose, the capsules were stored at 25 °C ± 2 °C under 60% ± 5% of relative humidity. Gram negative bile tolerant, *Escherichia coli*, *Salmonella* spp., *Staphylococcus aureus*, total yeast and mould, and total aerobic microbial counts were performed according to the European Pharmacopoeia and UNI EN ISO 6579–1:2017 methods. In addition, the viability of the *L. rhamnosus* TOM 22.8 strain encapsulated strain was monitored for 18 months thought viable cells count.

### Pilot-study design and patient enrolment

An observational pilot-study was conducted at the Department of General Surgery and Medical Surgical Specialties of the University of Catania, over a period of 1 year. Women in the reproductive age (18–45 years), attending to the Gynaecological Service of the aforementioned unit, were subjected to a preliminary gynaecological evaluation which included accurate anamnesis and pelvic examination. Inclusion criteria were: age between 18 and 45 years, presence of at least one vaginal sign or symptom (leucorrhoea, burning, itching and subjective vaginal discomfort), presence of at least 3 Amsel criteria^42^ and a Nugent score ≥ 7^43^ or a lactobacillary grade ≥ 2 (LBG), according to Donders classification^44^, presence of blastosporae and/or pseudohyphe evaluated by fresh mount microscopy. All the women presenting sexually transmitted disease due to *Chlamydia*, *Neisseria gonorrhoeae*, or *Trichomonas vaginalis* as well as those with specific cervico-vaginitis or severe vulvovaginal symptoms related to acute candidiasis; clinically apparent herpes simplex infection; human papillomavirus or human immunodeficiency virus infections; use of antibiotic, antifungal, probiotic or immunosuppressive drugs during the past four weeks; use of vaginal contraceptives and any others physiological or pathological conditions that could potentially interfere with the results of the study (pregnancy or breastfeeding, chronic diseases, neoplastic disease, diabetes, genital tract bleeding) were excluded.

Seventy (70) patients were screened and 30 of them satisfying the aforementioned inclusion and exclusion criteria were included in the study. All enrolled patients were allocated to one of three experimental groups: group A, vaginally treated; group B, orally treated; and group C, which was managed with an expectant strategy. Each woman assigned to A or B group was instructed to take 1 capsule/day for 10 consecutive days orally or topically, respectively. Each capsule contained 10 log cfu/g of the probiotic strain *L. rhamnosus* TOM 22.8.

All the women were subjected to a complete assessment in three scheduled appointments: at baseline (T0), 10 days after the start of the treatment (T1), and 30 days after the end of the oral or vaginal probiotic administration (T2).

During the whole study, each woman accurately recorded in a personal diary any potential adverse reaction or any use of other treatments for the entire duration of the observational period. In addition, they have been warned to promptly report to the investigator any potential worsening of symptomatology. In cases of significant discomfort, worsening of self-reported symptoms as well as any clinical occurrence of acute vaginitis, subjects were immediately excluded from the experimental observation and treated with conventional specific therapies.

The study was conducted according to the Helsinki Declaration (2000) of the World Medical Association and current standards of good clinical practice. Written informed consent was obtained from all participants before enrolment. The study protocol was approved by the local ethics committee (Comitato Etico Catania 1, Azienda Ospedaliero-Universitaria “Policlinico-Vittorio Emanuele” Catania, registration number 157/2019/PO).

### Vaginal discharge sample collection and processing

For each scheduled appointment (T0, T1, and T2), vaginal discharge was sampled from the lateral vaginal wall and the posterior vaginal fornix using sterile cotton-tipped swabs. Clinical criteria were evaluated as previously reported^45^. In addition, the presence of blastosporae and/or pseudohyphe was performed according to Donders et al.^7^.

Vaginal discharge, were collected using sterile cotton-tipped swabs filled with transport medium (Transystem Amies Medium Clear, Biolife, Milan, Italy), immediately transferred, under refrigerated conditions, to the Laboratory of Microbiology of the Department of Agriculture, Food and Environment, University of Catania (Catania, Italy), and subjected to microbiological counts using the agar media and conditions described by Pino et al.^45^. All analyses were performed in triplicate and results were reported as mean log cfu/ml and standard deviation.

### Statistical analysis

Data are shown as means ± standard deviation (SD) or as frequencies (percentages) for continuous and categorical variables, respectively. Comparison among groups were assessed with Kruskal–Wallis test and Chi-squared or Fisher's exact tests for continuous or categorical variables, respectively. A P value ≤ 0.05 was considered statistically significant.

Gene levels expression of COX-1, COX-2, IL-8, and IL-10 in differentiated human macrophages and microbiological data of vaginal swabs were subjected to one-way ANOVA followed by Tukey’s multiple comparison test. Differences were considered statistically significant at P ≤ 0.05. All statistical analyses were performed using SPSS Version 25.0 (Armonk, NY: IBM Corp.).

## Supplementary Information

Supplementary Information.
