# Supplementary material for: A clinical pilot study on the effect of the probiotic Lacticaseibacillus rhamnosus TOM 22.8 strain in women with vaginal dysbiosis
Source: Sci Rep. 2021 Jan 28;11:2592. doi: 10.1038/s41598-021-81931-z (PMC7843994; doi:10.1038/s41598-021-81931-z)
Supplement: Supplementary file 1 — Supplementary Information. [file 41598_2021_81931_MOESM1_ESM.docx]

**Scientific Reports**

**A clinical pilot study on the effect of the probiotic Lacticaseibacillus rhamnosus TOM 22.8 strain in women with vaginal dysbiosis**

**Alessandra Pino**1,+**, Agnese Maria Chiara Rapisarda**2,+**, Salvatore Giovanni Vitale**2,**, Stefano Cianci**3,**,Cinzia Caggia**1**, Cinzia Lucia Randazzo**1,*, **and Antonio Cianci**2

1 Department of Agricultural, Food and Environment, University of Catania, Santa Sofia street 100, 95123 Catania, Italy

2Department of General Surgery and Medical Surgical Specialties, University of Catania, 95123 Catania, Italy.

3 Department of Women, Child and General and Specialized Surgery, University of Campania "Luigi Vanvitelli", 80138 Naples, Italy.

[*cranda@unict.it](mailto:*cranda@unict.it)

+ these authors contributed equally to this work

**Supplementary Figure S1.** Clinical symptoms perception from patients allocated in A, B, or C group.

| Tested antibiotics | EFSA Breakpoints  (μg/ml)^a^ | TOM 22.8^b^ |
| --- | --- | --- |
| Gentamicin | 16 | <4^S^ |
| Kanamycin | 64 | 16^S^ |
| Streptomycin | 32 | 8^S^ |
| Tetracycline | 8 | 4^S^ |
| Erythromycin | 1 | 1^S^ |
| Clindamycin | 1 | 0.25^S^ |
| Chloramphenicol | 4 | 1^S^ |
| Ampicillin | 4 | 2^S^ |
| Metronidazole | NM | >600 µg/ml |
| Clotrimazole | NM | >256 µg/ml |
| Boric acid | NM | >10000 µg/ml |

^a^ Breakpoints proposed by the European Food Safety Authority (EFSA); ^b^ MICs determined by micro-dilution method; s: susceptible; NM: not mentioned in the EFSA guidelines.

**Supplementary Table S1.** Minimum inhibitory concentration (MIC) values, expressed as μg/ml, showed by the *L. rhamnosus* TOM 22.8 strain.
